# Supplementary material for: A tale of two connectivities: intra- and inter-subject functional connectivity jointly enable better prediction of social abilities
Source: Front Neurosci. 2022 Sep 1;16:875828. doi: 10.3389/fnins.2022.875828 (PMC9475068; doi:10.3389/fnins.2022.875828)
Supplement: Supplementary file 1 [file Data_Sheet_1.pdf]

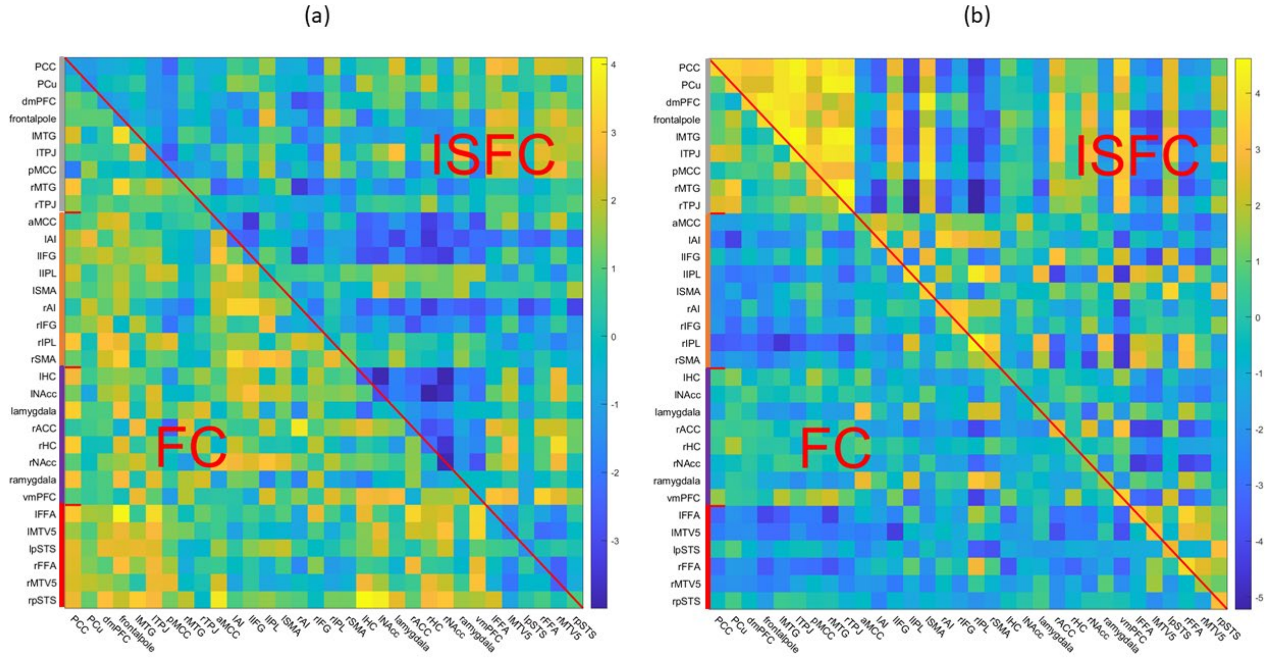

Figure S1. The two latent components identified in the final joint model. (a) The first latent factor explained 2.75% of the total connectivity variance, which mostly loaded onto the intra-subject neural coupling (FC). (b) The second latent factor explained 5.06% of the total connectivity variance, which loaded onto the inter-subject neural coupling (ISFC). The network assignment was color-coded based on the definition of (Alcalá-López et al., 2018). Grey: high-level processing; orange: intermediate-level processing; purple: limbic; red: visual-sensory.

Table S1. The thirty-two ROIs used in the study from the social brain atlas from (Alcalá-López et al., 2018). The parcellation is publicly available from the data-sharing platforms ANIMA (http://anima.fz-juelich.de/) and NeuroVault (http://neurovault.org/collections/2462/).

**Abbr.**	**Anatomical regions**	**MNI coordinates**			**Subnetwork**
		x	y	z	
PCC	Posterior cingulate cortex	-1	-54	23	high-level
PCu	Precuneus	-1	-59	41	high-level
aMCC	Anterior mid-cingulate cortex	1	25	30	intermediate
dmPFC	Dorsomedial prefrontal cortex	-4	53	31	high-level
frontalpole	Medial frontal pole	1	58	10	high-level
lAI	Left anterior insula	-34	19	0	intermediate
lFFA	Left fusiform gyrus	-42	-62	-16	visual-sensory
lHC	Left hippocampus	-24	-18	-17	limbic
lIFG	Left inferior frontal gyrus	-45	27	-3	intermediate

lIPL	Left inferior parietal lobe	-41	-41	42	intermediate
lMTG	Left middle temporal gyrus	-56	-14	-13	high-level
lMTV5	Left middle temporal V5 area	-50	-66	5	visual-sensory
lNAcc	Left nucleus accumbens	-13	11	-8	limbic
lSMA	Left supplementary motor area	-41	6	45	intermediate
lTPJ	Left temporo-parietal junction	-49	-61	27	high-level
lamygdala	Left amygdala	-21	-4	-18	limbic
lpSTS	Left posterior superior temporal sulcus	-56	-39	2	visual-sensory
pMCC	Posterior mid-cingulate cortex	Not provided			high-level
rACC	Rostral anterior cingulate cortex	-3	41	4	limbic
rAI	Right anterior insula	38	18	-3	intermediate
rFFA	Right fusiform gyrus	43	-57	-19	visual-sensory
rHC	Right hippocampus	25	-19	-15	limbic
rIFG	Right inferior frontal gyrus	48	24	2	intermediate
rIPL	Right inferior parietal lobe	54	-30	38	intermediate
rMTG	Right middle temporal gyrus	56	-10	-17	high-level
rMTV5	Right middle temporal V5 area	50	-66	6	visual-sensory
rNAcc	Right nucleus accumbens	11	10	-7	limbic
rSMA	Right supplementary motor area	48	6	35	intermediate
rTPJ	Right temporo-parietal junction	54	-55	20	high-level
ramygdala	Right amygdala	23	-3	-18	limbic
rpSTS	Right posterior superior temporal gyrus	54	-39	0	visual-sensory
vmPFC	Ventromedial prefrontal cortex	2	45	-15	limbic

Supplemental materials

On the relationship between FC, ISC and ISFC. By definition, we have

FC = corr(TC_sub1_ROI1, TC_sub1_ROI2) = corr(X, Y) = ρ_{XY}
ISC = corr(TC_sub1_ROI2, TC_sub2_ROI2) = corr(Y, Z) = ρ_{YZ}
ISFC = corr(TC_sub1_ROI1, TC_sub2_ROI2) = corr(X, Z) = ρ_{XZ}

For simplicity, the corresponding time courses are referred to as X, Y, and Z, and have zero mean and unit variance. Suppose we know FC (ρ_{XY}) and ISC(ρ_{YZ}), we can then express X and Z as follows

$$X = \rho_{XY}Y + O_Y^X \quad ,$$

$$Z = \rho_{YZ}Y + O_Y^Z \quad ,$$

where O_Y^X is the component of X that is orthogonal to Y (error). Then we can write ISFC (ρ_{XZ}) as

$$\rho_{XZ} = corr(\rho_{XY}Y + O_Y^X, \rho_{YZ}Y + O_Y^Z) = \rho_{XY}\rho_{YZ}\langle Y, Y\rangle + \langle O_Y^Z, O_Y^X\rangle + \rho_{XY}\langle Y, O_Y^X\rangle + \rho_{YZ}\langle Y, O_Y^Z\rangle \quad ,$$

where $\langle X, Y\rangle$ is the dot product of the two variables.

Recall that all three variables are of zero mean and unit variance, and both O_Y^X and O_Y^Z are orthogonal to Y, thus we can simplify ρ_{XZ} as

$$\rho_{XZ} = \rho_{XY}\rho_{YZ} + \langle O_Y^Z, O_Y^X\rangle \quad ,$$

Using Cauchy-Schwarz inequality, we can show that

$$\langle O_Y^Z, O_Y^X\rangle^2 \leq \langle O_Y^Z, O_Y^Z\rangle^2 \cdot \langle O_Y^X, O_Y^X\rangle^2 \quad ,$$

where $\langle O_Y^Z, O_Y^Z\rangle^2 = 1 - \rho_{YZ}^2$, and $\langle O_Y^X, O_Y^X\rangle^2 = 1 - \rho_{XY}^2$.

Thus, we can show that ISFC (ρ_{XZ}) is within a certain range depending on FC and ISC.

$$\rho_{XY}\rho_{YZ} - \sqrt[2]{(1-\rho_{XY}^2)1-\rho_{YZ}^2} \leq \rho_{XZ} \leq \rho_{XY}\rho_{YZ} + \sqrt[2]{(1-\rho_{XY}^2)1-\rho_{YZ}^2} \quad .$$
